# A case of renal diffuse large B-cell lymphoma concurrent with disseminated muscle involvement

**DOI:** 10.1186/s12882-025-04503-7

**Published:** 2025-10-07

**Authors:** Taha Enes Cetin, Remzi Goktug Yahyaoglu, Irem Saribiyik, Onur Ertunc, Omer Faruk Akcay, Yasemin Erten

**Affiliations:** 1 Department of Nephrology, Gazi University, Ankara, Turkey; 2 Department of Internal Medicine, Gazi University, Ankara, Turkey; 3 Department of Pathology, Gazi University, Ankara, Turkey

**Keywords:** Renal lymphoma, Acute kidney injury, Musculoskeletal invasion

## Abstract

**Background:**

Renal involvement in the diffuse large B-cell lymphoma (DLBCL) is rare and usually occurs as part of systemic disease. Muscle infiltration is even less common and can mimic inflammatory or obstructive conditions.

**Case presentation:**

We present a 58-year-old male with right flank pain, myalgia, oliguria, and weight loss. Initial evaluation suggested obstructive nephropathy due to ureteral stones, but renal function did not improve after stone removal. Urine cytology revealed atypical lymphoid cells positive for PAX-5. ^18^F-Flourodeoxyglucose Positron Emission Tomography- Magnetic Resonance Imaging (^18^F-FDG-PET-MRI) showed pathological ^18^F-FDG uptake in the kidneys, multiple muscles, and lymph nodes. Renal biopsy confirmed DLBCL. The patient was treated with daratumumab, rituximab, etoposide, cyclophosphamide, doxorubicin, vincristine, and prednisone, achieving hematologic remission, but remained dialysis-dependent.

**Conclusion:**

This case highlights an unusual presentation of secondary renal DLBCL with diffuse muscle involvement, mimicking obstructive acute kidney injury (AKI). The disease should be considered in atypical AKI presentations. Despite systemic treatment response, renal prognosis may be poor in advanced disease.

## Introduction

Lymphomas are capable of developing in almost all extranodal organs; however, renal involvement is considered one of the rarest manifestations. In most cases, kidney infiltration is part of disseminated nodal or extranodal disease, commonly referred to as secondary renal lymphoma [1]. Nevertheless, in exceptional cases, the kidney itself may serve as the primary site of disease origin, without evidence of systemic involvement, and is classified as primary renal lymphoma. Secondary renal involvement in Non-Hodgkin lymphoma (NHL) is relatively common in advanced, disseminated stages and can be seen in up to 30–60% of NHL patients [2].

Muscular involvement in lymphoma is an extremely infrequent occurrence. In such cases, the differential diagnosis can be complex, often requiring distinction from conditions like immune-mediated myositis or other infiltrative myopathies due to overlapping clinical and radiological feature [3]. Herein, we report a case of a 58-year-old male who initially presented with clinical findings suggestive of post-renal acute kidney injury (AKI) secondary to nephrolithiasis, but was ultimately diagnosed with bilateral renal diffuse large B-cell lymphoma (DLBCL) with concurrent disseminated muscular involvement.

## Case presentation

A 58-year-old male was admitted with right flank pain, extensive myalgia, decreased urine output, malaise, anorexia, pruritus, and an unintentional weight loss of 4 kg over the preceding six weeks. His medical history was notable for recurrent nephrolithiasis. He had a 45-pack-year smoking history but denied alcohol use. On physical examination, right costovertebral angle tenderness and mild (+ 1) pretibial edema were noted. Vital signs revealed a temperature of 36.8 °C and slightly elevated systolic blood pressure (154/78 mmHg), while other parameters were within normal limits. Laboratory investigations demonstrated elevated serum creatinine (4.64 mg/dL), blood urea nitrogen (59 mg/dL), potassium (6.1 mEq/L), uric acid (7.1 mg/dL), phosphate (4.1 mg/dL), and serum calcium (8.8 mg/dL), with a decreased estimated glomerular filtration rate (eGFR) of 14 mL/min/1.73 m². Additional findings included an increased LDH level (359 U/L), hemoglobin of 12.2 g/dL, white blood cell count of 9.67 × 10³/µL, neutrophil count of 6.61 × 10³/µL, and platelet count of 340 × 10³/µL (Table 1). A 24-hour urine collection revealed 950 mg/day of proteinuria, predominantly non-albumin in nature. Urinalysis revealed 2 erythrocytes and 9 leukocytes per high-power field. Ultrasonography of the urinary tract showed enlarged kidneys, measuring 138 × 67 mm on the left and 131 × 66 mm on the right, with diffusely increased parenchymal thickness. The right kidney exhibited grade 2 hydronephrosis, and a 6 mm echogenic focus was detected in the proximal right ureter, consistent with a ureteral calculus. The urine culture was sterile. The patient subsequently underwent ureterorenoscopy (URS) with Double-J stent (DJS) placement, resulting in the successful removal of the stone. Despite the intervention, urine output did not increase, and intermittent hemodialysis was initiated due to persistent uremic symptoms. Autoimmune serologies, including antinuclear antibody, antineutrophil cytoplasmic antibody (ANCA), complement component 3 (C3), C4, kappa, lambda light chains, and anti-glomerular basement membrane antibodies, were all negative.

**Table 1:** Admission blood and urine tests

Test	Result	Reference Range
Blood Urea Nitrogen (BUN)	59	7–20 mg/dL
Creatinine	4.64	0.7–1.3 mg/dL
Glomerular Filtration Rate (GFR)	14	> 90 mL/min/1.73 m²
Albumin	4.3	3.5–5.0 g/dL
Bilirubin, Total	0.28	0.1–1.2 mg/dL
LDH	359	140–280 U/L
Sodium	140	135–145 mmol/L
Potassium	6.1	3.5–5.1 mmol/L
Uric acid	7.1	3.4-7 mg/dL
Phosphate	4.1	2.5–4.5 mg/dL
Calcium	8.8	8.6–10 mg/dL
Hemoglobin (HGB)	12.2	13.5–17.5 g/dL (male)
White Blood Cell (WBC)	9.67 × 10³	4.5–11.0 × 10³/µL
Neutrophil Count	6.61 × 10³	2.0–7.0 × 10³/µL
Platelet count	340	150–400 × 10³/µL
Erythrocyte Sedimentation Rate (ESR)	69	0–20 mm/h
C-Reactive Protein (CRP)	13.1	0–5 mg/L
Albumin/Creatinine (Spot Urine)	260 mg/g	< 30 mg/g
Protein/Creatinine (Spot Urine)	950 mg/g	< 200 mg/g
Urine Erythrocytes	2	0–3 high-power field
Urine Leukocytes	9	0–10 high-power field
Creatine kinase	44	38–174 U/L

**Fig. 1:**
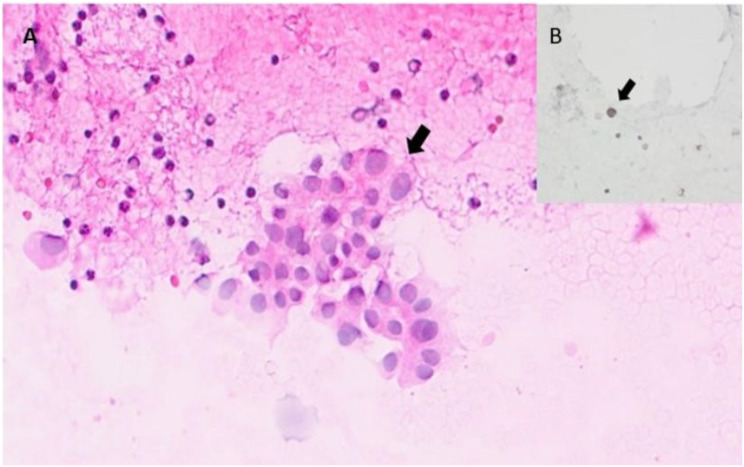
Urine cytology showed normal urothelial and lymphoid cells. Atypical lymphoid cells, more than twice the size of normal lymphocytes and with nuclear irregularities, were observed in a few areas. Atypical cells showed nuclear staining with Pax-5. **(A)** Urine cytology showing large atypical lymphoid cells (H&E, ×400) **(B)** Atypical cells positive with Pax-5 immunohistochemistry (×400). Findings are indicated with arrows

**Fig. 2:**
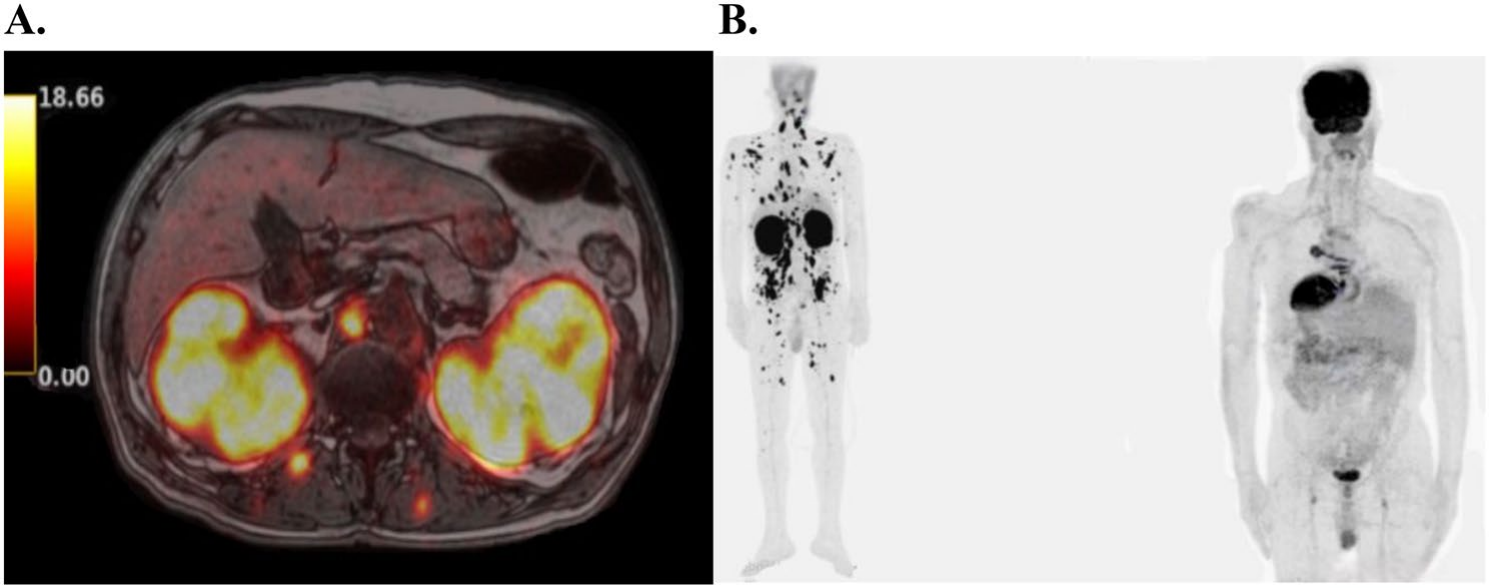
**(A)** Axial section PET/MR imaging at the T12 vertebral level demonstrates lobulated contours with bilateral renal involvement. Focal increased ^18^F-FDG uptake in the kidneys and surrounding soft tissues **(B)** The pre-treatment Maximum Intensity Projection (MIP) image demonstrates nodular/diffuse patterns of increased ^18^F-FDG uptake in the trunk, proximal extremities, and bilateral kidneys. The post-treatment MIP image revealed that the distribution of the radiopharmaceutical in all other visualized body regions and bone structures was consistent with physiological distribution

**Fig. 3:**
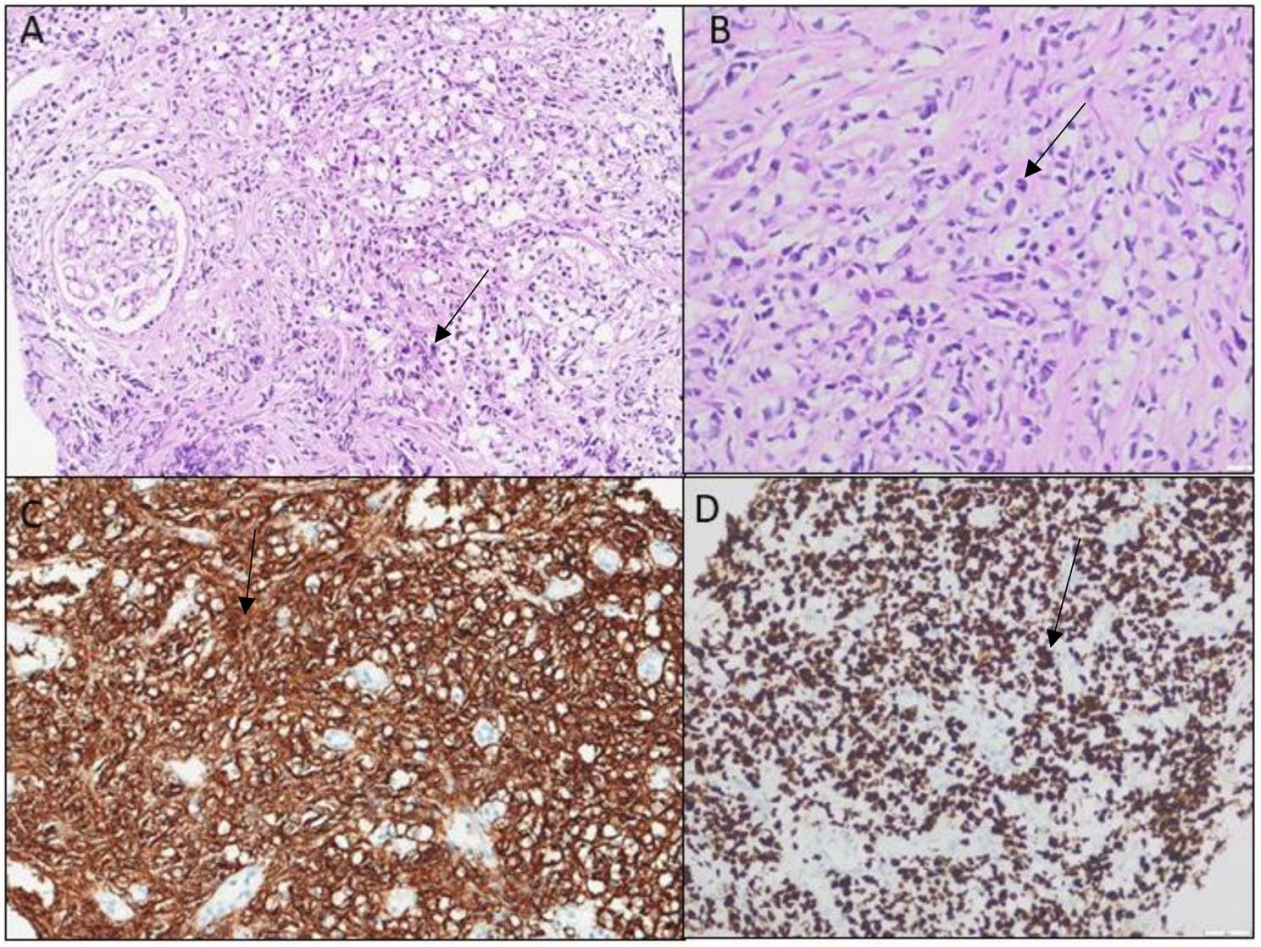
Kidney biopsy findings**.** Normal glomeruli are observed. An infiltration composed of small and large atypical lymphoid cells obliterates the normal renal parenchyma. In immunohistochemical staining, large atypical cells are positive for CD20, Pax5, and Bcl-6, but negative for MUM1, Bcl-2, Cyclin D1, Myc, and TdT. (**A**) Infiltrating mixed lymphoid cells in renal parenchyma (hematoxylin and eosin, ×200). (**B**) Large atypical lymphoid cells (H&E, ×400). (**C**) Positive with CD20 immunohistochemistry (×200). (**D**) High proliferative activity with Ki-67 staining (×200). Findings are indicated with arrows

Due to bilateral renal enlargement and sterile pyuria, urine cytology was performed and revealed atypical lymphoid cells exhibiting nuclear irregularities and PAX-5 positivity, raising suspicion for an underlying lymphoma (Fig. 1). Subsequent ^18^F-Flourodeoxyglucose Positron Emission Tomography-Magnetic Resonance (^18^F-FDG-PET-MR) imaging demonstrated pathological ^18^F -FDG uptake in multiple sites, including the thyroid gland, mediastinal lymph nodes, paraspinal and abdominal muscles, and myocardium, along with bilateral renal parenchymal involvement (Fig. 2). Normal serum levels of creatine kinase and myoglobin made inflammatory myopathy less likely in the differential diagnosis. In the light of persistent renal dysfunction and abnormal urine cytology, a renal biopsy was performed, which confirmed the diagnosis of DLBCL (Fig. 3). The patient was initiated on a combination chemotherapy regimen of daratumumab, rituximab, etoposide, cyclophosphamide, doxorubicin, vincristine, and prednisone (Da-R-CHOEP). Following treatment, complete regression of lymphomatous infiltration was achieved, as also demonstrated in the post-treatment Maximum Intensity Projection (MIP) image (Fig. 2B), which showed only physiological distribution of radiopharmaceutical uptake. However, despite hematologic remission, renal function did not recover, and the patient remained dialysis-dependent.

## Discussion

The case initially resembled a typical postrenal AKI secondary to obstructive uropathy. However, the initial symptoms of the patient, including nephrolithiasis, oliguria, diffuse myalgia, and unintentional weight loss, alongside progressive renal dysfunction after stone removal, posed a substantial diagnostic challenge. Although the obstructive component was managed with URS and DJS placement, persistent AKI and ongoing uremic symptoms necessitated further evaluation. Pathologic examination of renal biopsy ultimately confirmed DLBCL, characterized by extensive renal parenchymal infiltration. PET-MR imaging demonstrated significant FDG uptake in bilateral renal, ureteral, and lymph node involvement, supporting a diagnosis of secondary renal DLBCL.

Secondary renal lymphoma accounts for less than 3% of extranodal lymphomas [4]. In secondary renal lymphoma, the tumor infiltrates kidneys mainly via lymphatic vessels. It has been proposed that this process begins in the capsule, subcapsular tissue, or renal sinus, subsequently extending into the renal parenchyma. Tumor proliferation typically occurs within the interstitium, involving adjacent nephrons and collecting ducts, with blood vessels providing a structural framework for tumor expansion. This infiltrative growth pattern often preserves the renal contour and underlying parenchymal architecture [5].

Additionaly, existence of disseminated skeletal muscle involvement further complicated the clinical picture, necessitating differentiation from inflammatory myopathies, such as myositis, and other infiltrative muscle disorders. The incidence of muscular involvement in lymphoma is approximately 0.4% in Hodgkin lymphoma and 1.5% in non-Hodgkin lymphoma [6]. This involvement is typically a result of contiguous spread from adjacent lymph nodes or primary bone lesions [7]. In addition, lymphoproliferative diseases with high tumor burden may predispose to metabolic abnormalities such as hyperuricemia and hyperphosphatemia, which can contribute to de novo nephrolithiasis and aggravate renal injury, even in the absence of overt tumor lysis syndrome [8].

In the presence of unexplained acute kidney injury and enlarged kidneys, renal lymphoma should be considered, and kidney biopsy remains the gold standard for its diagnosis. In this case, DLBCL was initially suspected based on urine cytology and subsequently confirmed through histopathological examination. For aggressive lymphomas such as DLBCL, systemic chemotherapy is the cornerstone of treatment, while surgical intervention is generally avoided due to the associated morbidity and mortality [9]. The CHOP regimen is commonly used, either with or without rituximab, and has demonstrated improved outcomes [9]. Previous studies have reported that addition of daratumumab to rituximab-based regimens has yielded promising results in the treatment of DLBCL [10]. Our patient was treated with the daratumumab-R-CHOEP regimen. Despite an effective hematologic response, function of the patient did not improve, resulting in end-stage kidney disease and requiring dialysis.

To the best of our knowledge, this is the first reported case of secondary renal DLBCL presenting with diffuse muscle infiltration. This case emphasizes the importance of considering hematologic malignancies in patients with atypical AKI presentations, particularly when imaging and cytological findings raise suspicion. Early recognition and histopathological confirmation are crucial for appropriate management, although renal prognosis may remain poor in advanced-stage disease.

## Data Availability

All data supporting the conclusions of this article are included within the manuscript.

## Abbreviations

NHL: Non-Hodgkin lymphoma
AKI: Acute kidney injury
DLBCL: Diffuse large B-cell lymphoma
eGFR: Estimated glomerular filtration rate
URS: Ureterorenoscopy
DJS: Double-J stent
ANCA: Antineutrophil cytoplasmic antibody
C3: Complement component 3
^18^F-FDG: ^18^F-Fluoro-deoxyglucose
^18^F-FDG-PET-MR: ^18^F-Fluoro-deoxyglucose Positron Emission Tomography–Magnetic Resonance
Da-R-CHOEP: Daratumumab, rituximab, etoposide, cyclophosphamide, doxorubicin, vincristine, and prednisone
MIP: Maximum Intensity Projection
